# (Bio)degradable Biochar Composites of PLA/P(3HB-*co*-4HB) Commercial Blend for Sustainable Future—Study on Degradation and Electrostatic Properties

**DOI:** 10.3390/polym16162331

**Published:** 2024-08-17

**Authors:** Marta Musioł, Joanna Rydz, Henryk Janeczek, Jacek Andrzejewski, Mariana Cristea, Krzysztof Musioł, Marian Kampik, Marek Kowalczuk

**Affiliations:** 1 Centre of Polymer and Carbon Materials, Polish Academy of Sciences, M. Curie-Skłodowska 34 St., 41-819 Zabrze, Poland; jrydz@cmpw-pan.pl (J.R.); hjaneczek@cmpw-pan.pl (H.J.); mkowalczuk@cmpw-pan.pl (M.K.); 2 International Polish-Romanian Research Laboratory ADVAPOL, Centre of Polymer and Carbon Materials, Polish Academy of Sciences, M. Curie-Skłodowska 34 St., 41-819 Zabrze, Poland; 3 Institute of Materials Technology, Polymer Processing Division, Faculty of Mechanical Engineering, Poznań University of Technology, Piotrowo 3 St., 61-138 Poznań, Poland; jacek.andrzejewski@put.poznan.pl; 4 “Petru Poni” Institute of Macromolecular Chemistry, 41-A. Aleea Gr. Ghica Voda, 700487 Iasi, Romania; mcristea@icmpp.ro; 5 International Polish-Romanian Research Laboratory ADVAPOL, “Petru Poni” Institute of Macromolecular Chemistry, 41-A. Aleea Gr. Ghica Voda, 700487 Iasi, Romania; 6 Department of Measurement Science, Electronics and Control, Silesian University of Technology (SUT), Akademicka 10 St., 44-100 Gliwice, Poland; krzysztof.musiol@polsl.pl (K.M.); marian.kampik@polsl.pl (M.K.); 7 Wolverhampton School of Sciences, Faculty of Science and Engineering, University of Wolverhampton, Wulfruna Street, Wolverhampton WV 11 LY, UK

**Keywords:** biodegradable composites, PLA, P(3HB-*co*-4HB), biochar, electrostatic properties, composting

## Abstract

Interesting alternatives to expensive biodegradable polymers are their composites with natural fillers. The addition of biochar to a blend of poly(lactic acid) (PLA) and poly(3-hydroxybutyrate-*co*-4-hydroxybutyrate) was studied, and the resulting materials were evaluated for their properties and changes during degradation. Introducing biochar as a filler brought a noticeable improvement in electrostatic properties. Surface resistivity decreased from 3.80 × 10^12^ for the sample without biochar to 1.32 × 10^12^ for the sample with 30% filler content. Degradation tests revealed distinct differences in the degradation profile for composites due to the presence of filler. Composites with a lower biochar content displayed curling crack edges during hydrolytic degradation, and when the filler content reached 20 wt%, PLA loss accelerated. This study suggests that biochar-based composites have potential to be used as sustainable materials with improved properties.

## 1. Introduction

The growing awareness of society regarding the impact of our daily decisions on changes in the natural environment increases the demand for products that ensure an adequate standard of living and comfort while limiting their negative impacts on the natural environment. According to the EU’s Plastics Strategy, the new vision includes an approach whereby by 2030, packaging made of plastics introduced to the EU market should be recycled in a way that is economically justified or suitable for reuse. A gate is opened for the growth of the market of biodegradable polymers perceived as innovative materials obtained from alternative renewable sources that can replace those of non-renewable origin [1]. Plastic products with a limited lifespan, so widely used, are increasingly being made from biodegradable polymers. Among these materials, the most popular include poly(lactic acid) (PLA), polyhydroxyalkanoates (PHAs), poly(butylene succinate) (PBS), and poly(1,4-butylene adipate-*co*-1,4-butylene terephthalate) (PBAT). The Biodegradable Polymers Global Market Report 2022–2030 indicates that PLA, mainly due to its technological maturity, is at the center of interest of both researchers and industry. However, it is still a young market and struggling with problems resulting from the high prices of its products as well as a limited amount of infrastructure enabling its organic recycling. Market growth boosts are expected, especially in the packaging and textile industries, caused by government regulations and public awareness [2]. The applications of biodegradable polymers sometimes require special properties, which, however, PLA itself may not have. This increases the possibilities of developing the technology of other biodegradable polymers, which, being a component of a blend with PLA or an additive, will change the properties of the finished product without limiting its main advantage, which is biodegradability [3,4]. The blending of PLA with, among others, poly(3-hydroxybutyrate-*co*-4-hydroxybutyrate) (P(3HB-*co*-4HB)) is a simple and effective method for improving strength and processability. P(3HB-*co*-4HB) belongs to the polyesters produced by bacteria [5]. The presence of more flexible 4HB units in the copolymer causes changes in mechanical properties. The amount of variation depends on its content [6]. The properties of P(3HB-*co*-4HB) and the possibility of obtaining a blend with PLA by simple methods cause a significant increase in interest in both the copolymer itself and its blends [7,8,9,10].

Bioplastics, made from renewable biomass sources, can help mitigate the environmental impacts of conventional plastics. Additionally, biodegradable bioplastics can break down naturally in the environment, reducing the amount of plastic waste that ends up in landfills and oceans. The biodegradation of PHA by microorganisms occurs in both aerobic and anaerobic environments. Aerobically, biodegradation yields biomass, carbon dioxide (CO_2_), and water (H_2_O) as harmless end products, while anaerobically, it produces biomass, CO_2_, methane, and H_2_O. This natural process is non-hazardous to the environment and is an essential part of the ecosystem’s nutrient cycle [11]. The biodegradation of PLA occurs through a two-step process. In the first step, heat and moisture in the compost environment cause PLA chains to break down into smaller molecules, resulting in the formation of lower molecular weight oligomers. Microorganisms can utilize shorter polymer chains, breaking them down further into smaller oligomers and monomers, which are then metabolized into H_2_O and CO_2_ [12].

Niche applications of this type of polymer do not mainly focus on the price but on the potential of the material. However, price reduction opportunities may be crucial overall. An interesting alternative in this area is the use of fillers, which together with the polymer form a composite; this leads to a reduction in price and modifies the properties of the material in relation to the polymer itself [4,13]. For composites in which the matrix consists of biodegradable polymers, selecting appropriate fillers is of particular importance in ensuring the biodegradability of the final material. As has already been described, a lot of interest in this area is caused by biomass. However, its sensitivity to water limits its use as filler. The use of pyrolysis for biomass modification has a number of advantages and allows biochar with appropriate properties to be introduced as a filler into biodegradable composites. The comparison indicates that the costs of talc or chalk, commonly used as commercial additives, are roughly equivalent to that of biochar, suggesting that biochar can be a viable alternative without significant financial disadvantages in applications where such additives are required. The use of biochar as a filler in composites additionally modifies its electrostatic properties [14]. Lowering the resistivity of products in which biochar is used as a filler significantly increases their application possibilities. Products with appropriate parameters can serve as packaging for, e.g., electronic components [15]. The increase in the conductivity of the biodegradable material caused by the presence of the conductive filler also leads to the increased absorption of electromagnetic waves. As a result of this, there is the possibility to obtain high-performance electromagnetic interference shielding biodegradable materials [16]. In the case of packaging, for example, food with a longer use-by date, external appearance is important. Plastic product packaging on store shelves easily attracts dust due to its insulating properties by the creation of static charges. The use of a conductive filling should result in the antistatic properties of the packaging, which definitely improves the visual perception of the product by the consumer [17].

Recent studies have shown that both the filler content and the type of matrix play crucial roles in determining the surface resistivity of biochar composites. Specifically, composites with matrices containing varying proportions of PLA and PBAT exhibited optimal properties when the PLA content was maximized, highlighting the importance of material selection in enhancing the electrical performance of these composites [14,18].

Depending on the planned end use of the product, its electrostatic properties can be manipulated by selecting the appropriate amount of filler and the type of polymer in composites. In this research, PLA/P(3HB-*co*-4HB) was used as a matrix, and the biochar filler was added in amounts of 10, 15, 20, and 30 wt% for the preparation of composites. Previously published mechanical tests were carried out for developed materials, which indicated that the addition of biochar in composites was found to have a significant impact on the properties determined in tensile tests. Notably, as the biochar concentration increased, the elongation at break decreased, indicating a decrease in flexibility. The stiffness of the composites also increased with a higher biochar content, making the material more rigid. Additionally, the tensile strength decreased with increasing biochar concentration, while the viscosity of the material increased due to the matrix-filler interactions slowing down the mobility of polyester chains. The lack of significant changes in the FTIR spectra of the investigated materials suggested that the addition of biochar does not alter the overall composition dramatically. However, the decrease in the intensity of polymer matrix bands with increased biochar content in the composite was observed. The SEM analysis of the fracture surfaces for both composites revealed the presence of biochar filler particles, with the 30 wt% biochar composite exhibiting a predominance of these particles [19]. Changing the matrix of composites with biochar from PBAT/PLA used in previous studies [14] to PLA/P(3HB-*co*-4HB) made it possible to assess the impact on the electrostatic properties and not only the amount of filler but also the use of a different matrix. The application of a specific material and modifications of its properties are very important, but they cannot be forgotten at the end of the product’s life cycle. The assumption for products made of biodegradable polymers is their organic recycling when they become waste.

## 2. Materials and Methods

### 2.1. Materials

As a matrix, SOOGREEN 2001a (PLA/P(3HB-*co*-4HB)) (Tianjin GuoYun Biological Material Co. Ltd., Tianjin, China) was used, which is a commercial blend of 78 mol% PLA with 21 mol% P(3HB-*co*-4HB) (containing 12 mol% 4-hydroxybutyrate (4HB) units) and 1 mol% plasticizer acetyl tributyl citrate (ATBC), as determined by NMR analysis. ATBC is an efficient plasticizer, lowering the glass transition temperature and increasing PHA’s ability to thermoplasticize [20]. Biochar from Fluid S.A. (Sędziszów, Poland) was employed as filler. The material in the form of unprocessed powder with a very wide range of particle sizes was pre-sieved to eliminate large fragments of wood impurities. Next, the biochar filler was ball-milled, which allowed us to obtain particles with an average size of approximately 1 μm. The ball milling procedure was conducted for 24 h. The method of obtaining was explained in detail elsewhere [14,21]. Before the following processing, the biochar filler was dried at 100 °C for 24 h (vacuum drier) to prevent excessive moisture content.

### 2.2. Preparation of Composites

The melt blending procedure was conducted using the lab-scale co-rotating twin-screw extruder model EH16.2D (Zamak Mercator, Skawina, Poland). Before extrusion, the PLA/P(3HB-*co*-4HB) pellets were dried using a cabinet drier (60 °C/24 h). The pre-mixing of the pellets and powder filler was conducted using a rotary mixer; the prepared dry-blend was then transferred to the extruder hopper. The material was fed volumetrically to the extruder barrel. The screw speed was 100 rpm, while the temperature profile was set as follows: 160 (die)–165–170–170–170–165–165–160–100 (hopper) °C. The extrusion rate under these conditions was about 2 kg/h. The extrusion parameters were similar for all of the compounded materials, where the concentrations of the biochar filler were 10, 15, 20, and 30 wt%. The extruder materials were cooled down, pelletized, and then stored in a cabinet drier before the injection molding process (60 °C/24 h).

The molding procedure was conducted using an injection molding press, model Engel e-mac 50 (Engel GmbH, Schwertberg, Austria). The injection molding procedure was conducted at 175 °C, measured at the nozzle. The injection and holding pressures were set to 1000 and 600 bar, respectively. The holding time was 15 s, and cooling time was 45 s. The mold temperature was 40 °C. The molding procedure allowed us to obtain dumbbell samples (type 1A according to ISO 527 standard [22]).

### 2.3. Characterization Studies

#### 2.3.1. Thermal Properties

The thermal stability of the investigated samples was evaluated using thermogravimetric analysis (TGA) under a nitrogen atmosphere with a heating rate of 10 °C/min by TGA/DSC1 Mettler-Toledo (Columbus, OH, USA).

Dynamic mechanical analysis (DMA) tests were carried out on an RSA G2 (TA Instruments), in bending mode (three-point bending clamp). The isochronal experiments (1 Hz) were run with a heating rate of 2 °C/min, from −100 °C up to the temperature at which the sample was no longer load-bearing. The bar samples with dimensions of 25 mm × 10 mm × 4 mm were deformed with a 0.01% strain, which was well within the viscoelastic linear range. 

The differential scanning calorimetry (DSC) study for neat samples was performed using a Discovery DSC 250 (TA Instruments, New Castle, DE, USA) at a heating/cooling rate of 20 °C/min, between −100 and 200 °C, under a nitrogen atmosphere (purge rate 50 mL/min). The thermal parameters of samples after incubation in a degradation environment were analyzed using the DSC Q2000 apparatus (TA Instruments, New Castle, DE, USA). The experiment was conducted under a nitrogen atmosphere at a flow rate of 50 mL/min at a heating rate of 20 °C/min. The first heating run in which the thermal history was suppressed was taken from −80 °C to 200 °C. The glass transition temperature (*T_g_*) was determined for the amorphous sample (obtained by quenching the melted samples from melt (200 °C)) in the second heating run from −80 °C to 200 °C.

#### 2.3.2. Visual Examination

Scanning electron microscopy (SEM) visualized the surface of the tested sample. A Quanta 250 FEG (FEI Company, Fremont, CA, USA) high-resolution environmental scanning electron microscope was used for this purpose. Pictures were obtained for the samples placed under a low vacuum (80 Pa), and an acceleration voltage equal to 10 kV was used. The samples were placed on carbon tape without additional preparation.

#### 2.3.3. Surface Resistivity Measurement

Surface resistivity measurement was conducted by a two-terminal test circuit with a nanoammeter V623 (Meratronik, Warsaw, Poland) and voltmeter V560 (Meratronik, Warsaw, Poland) according to International Standard IEC 62631-3-2:2015 [23], as described elsewhere [14]. Line electrodes provided by two parallel lines, separated by a gap, were applied to the test specimen’s surface using a conductive material. Silver paint was used to apply electrodes on the sample surfaces. In this type of measurement, a very stable voltage source is required, which is why a high-performance direct voltage calibrator Fluke 5440A (Fluke Corporation, Washington, DC, USA), was used; the set voltage was 100 V.

The temperature during the measurements was 23 °C, and the relative humidity of the air was 50%. The voltage (*V*) and current (*I*) values were measured, and then the surface resistance (*R_s_*) was calculated using Ohm’s law.

Then surface resistivity *σ* was calculated from Equation (1)

(1) $$\sigma =\frac{l}{g}\cdot R_{s}$$

where

*l*—length of line electrodes;
*g*—distance between the lines (gap);
*R_s_*—surface resistance.

Two research objects were made of each material, for which three measurements were carried out, so the final result is the average of 6 measurements. 

#### 2.3.4. Nuclear Magnetic Resonance (NMR) Measurements

^1^H NMR spectra were recorded using a Bruker-Advance spectrometer (Fremont, CA, USA). Each spectrum was collected with 64 scans, 11 µs pulse width, and 2.65 s acquisition time. The spectrometer operated at 600 MHz using tetramethylsilane (TMS) as the internal standard and CDCl_3_ as the solvent.

### 2.4. Degradation Environments

#### 2.4.1. Test under Composting Conditions

A biodegradation experiment in laboratory composting conditions was conducted for 21 days in a Micro-Oxymax respirometer Columbus Instruments S/N 110315 (Columbus, OH, USA) [24]. The compost used for the degradation test in the respirometer was donated by Station of Mechanical-Biological Waste Treatment in Zabrze, Poland. A total of 500 g of mature compost at a humidity level of 57% in 2 L jars was incubated at 58 °C. Samples were placed in the middle of the compost height. 

#### 2.4.2. Abiotic Degradation

For the hydrolytic degradation experiment in water, specimens were obtained from a neat blend, and all types of composites were cut into elements so that the average mass of each was 0.8 g. Samples prepared in this way were placed in 25 mL of demineralized water in screw-capped vials at 70 °C in the laboratory incubator (Mini-incubators of 5.4 lt – ICT 5.4, FALC, Treviglio, Italy). The research methodology was developed in accordance with the International Standard ISO 13781 [25], as previously described [26]. The 70-day incubation time was divided into periods after which the samples were removed from the medium, washed, and then dried to constant mass.

## 3. Results

The blending of PLA and P(3HB-*co*-4HB) is usually conducted to diminish the intrinsic brittleness of PLA due to the good flexibility of P(3HB-*co*-4HB) [27,28]. In addition, it is well established that a higher content of 4HB in the copolymer raises its flexibility [29]. Nevertheless, blending does not necessarily result in ductile PLA because the morphology also governs the properties of the blend. In this sense, different strategies have been used to improve the compatibility between PLA and P(3HB-*co*-4HB), like uniaxial stretching or melt blending in a specific temperature window [30,31].

**Figure 1:**
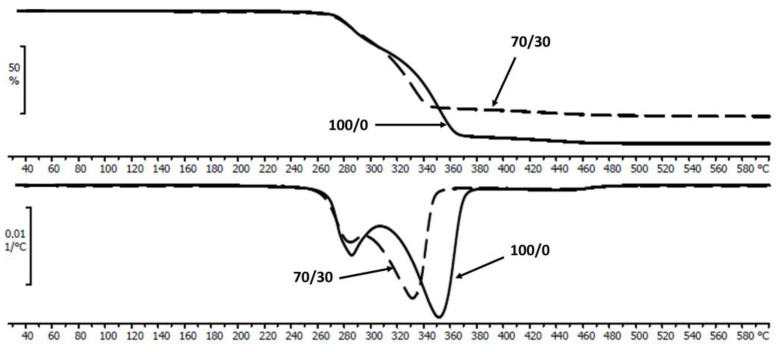
Thermal decomposition (TGA) curves and their first-order derivative (DTG) curves of the neat PLA/P(3HB-*co*-4HB) (100/0) and PLA/P(3HB-*co*-4HB)/biochar (70/30) before degradation.

TGA analysis was used to determine the thermal stability of the investigated materials before degradation tests. An evaluation of the thermal decomposition shows multiple mass loss steps that correspond to single materials of the blend. The temperature of the maximum decomposition peak around 280 °C was assigned to P(3HB-*co*-4HB) [32] and the second one around 350 °C to PLA [33]. Additionally, a small and wide peak between 400 °C and 460 °C was observed. It may come from impurities contained in P(3HB-*co*-4HB) after its manufacture [34] and/or from commercial stability-enhancing additives [35,36]. Figure 1 shows the thermal decomposition curves and their first-order derivative of the neat blend and its composite with 30 wt% of biochar. The presence of biochar in the PLA/P(3HB-*co*-4HB) matrix resulted in a decrease in the maximum decomposition temperature of the PLA contained in the blend. This phenomenon may result from the increase in the amount and dispersion of biochar and hence better heat transfer which for PLA increases with an increase in the orientation and crystallization of the molecular chains and stronger molecular chain interactions [37]. In the area which corresponds to the thermal decomposition of P(3HB-*co*-4HB), for composites, the *T_max_* value is not much different from the value for the neat matrix [38].

The TGA experiments were also performed to determine the onset of the degradation temperature of the samples, which represents the upper temperature limit of the DSC experiment. All the samples were stable enough up to 200 °C. Therefore, the DSC experiments were carried out safely until 200 °C.

**Figure 2:**
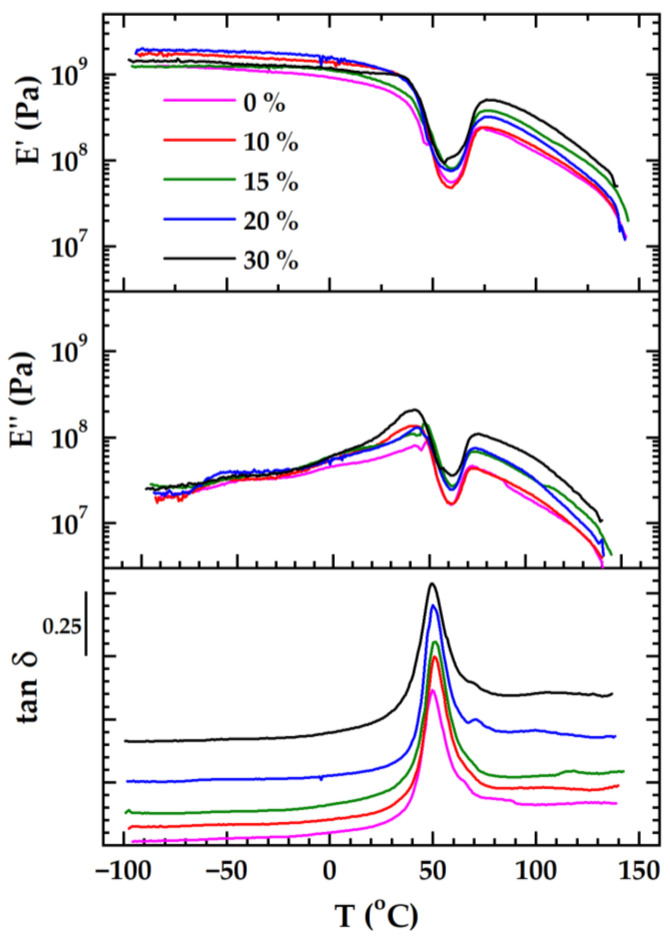
The variation in *E*′, *E*″, and tan *δ* for the neat PLA/P(3HB-*co*-4HB) (100/0) and PLA/P(3HB-*co*-4HB)/biochar composites with mass ratios of 90/10, 85/15, 80/20, and 70/30 at 2 °C/min and 1 Hz. The tan *δ* curves of composites were translated vertically to avoid overlapping.

The variation in the elastic modulus (*E*′), viscous modulus (*E*″), and loss factor (tan *δ*) with temperature is represented in Figure 2. For the sake of clarity, the tan *δ* curves of the samples with biochar (10, 15, 20, and 30 wt%) were translated vertically. The main viscoelastic characteristics are included in Table 1.

*E***′** is higher than *E*″ all over the temperature range investigated. In the glassy region, the values of the *E***′** modulus indicate that the presence of biochar increases the rigidity of the PLA/P(3HB-*co*-4HB) matrix as compared to the blend without filler (100/0), however not in a monotonous way. This aspect reflects the possibility that not all composites have a reinforcement effect. The filler could hamper the physical interactions between polymer chains and keep them apart, decreasing the cohesion of the material [39]. A recent review reported more accounts related to the effect of fillers on the viscoelastic behavior of PLA [40]. Additionally, in the glassy region, the secondary relaxation of PLA is perceptible on the *E*″ curves, centered from −50 to −60 °C [28,41].

**Figure 3:**
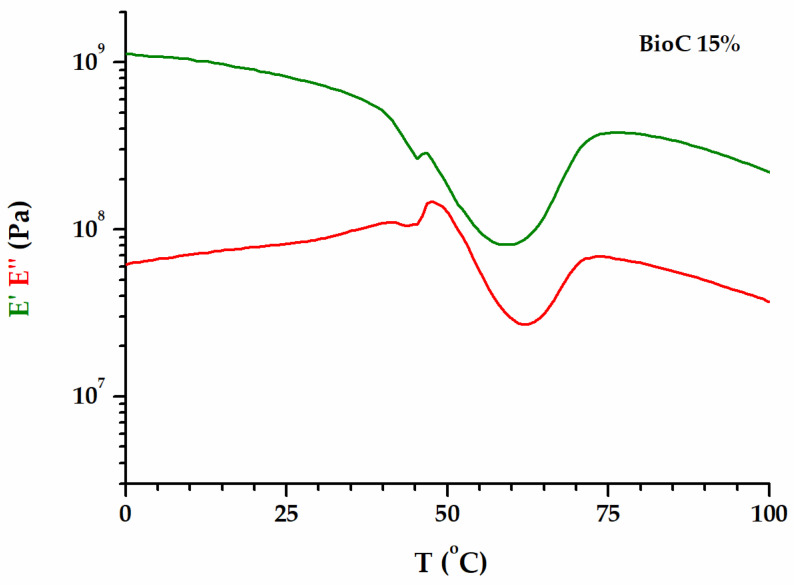
The variation in *E*′ and *E*″ in the glass transition region for PLA/P(3HB-*co*-4HB)/biochar (85/15).

The neat PLA and the blends/copolymers display unique viscoelastic behaviors among classic polymers. The pattern formed by the dependence of the elastic modulus *E*′ on the temperature offers a perspective of the complex phenomena that occur during the glass transition [28]. Figure 3 zooms in on the glass transition region for PLA/P(3HB-*co*-4HB)/biochar (85/15). 

As the macromolecular chains gain mobility in the region of the glass transition, the stiffness reduction is reflected in the decrease in the *E*′ modulus. However, a break in the *E*′ curve occurs, and a double peak is noticed in the *E*″ curve. This is consistent with the beginning of cold crystallization during heating through the glass transition temperature. A small diminution of mobility is registered at the emergence of crystalline domains. Still, the main α-relaxation process is dominant until the *E*′ modulus turns upward because the crystallization phenomenon takes over. Most often, the temperature of the tan *δ* peak is taken as the glass transition temperature, as it reflects the middle of the glass transition range for polymers. A well-behaved tan *δ* peak associated with an α-relaxation has a specific trend in the situation of a multifrequency experiment [42,43,44]. Briefly, the tan *δ* peak shifts to a higher temperature, and its height diminishes with increasing frequency. The glass transition temperatures for the investigated blends were estimated to be the onset of the elastic modulus *E*′ (*E*′_onset_). These values, along with the temperatures and the *E*′ moduli at which the strengthening effects of crystallization become more important than the mobility enhancement as a result of relaxation, are included in Table 1.

A higher quantity of biochar favors cold crystallization and raises the rigidity of the rubbery plateau. Nonetheless, it is important to emphasize the significant decline in the *E*′ modulus in this region, as opposed to neat PLA that has a stable rubbery plateau almost up to the melting temperature [28]. 

**Figure 4:**
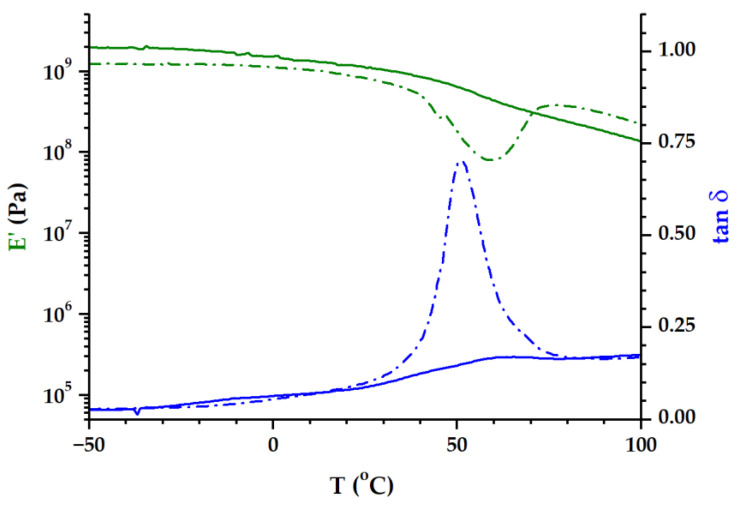
*E*′ modulus and tan *δ* plots for PLA/P(3HB-*co*-4HB)/biochar (85/15): first heating run (dotted line), second heating run (continuous line).

The observed DSC behavior of the PLA/P(3HB-*co*-4HB)/biochar composites during the second heating run was confirmed in DMA by performing a DMA experiment on a sample that was already subjected to temperature scanning DMA. Figure 4 comparatively presents the DMA results during the first and second heating for PLA/P(3HB-*co*-4HB)/biochar (85/15).

The cooling run was performed at a lower rate than the DSC cooling, and a higher quantity of the semi-crystalline phase was formed. This is reflected in the less steep decline in the *E′* modulus and the flattened tan *δ* peak in the second heating run. Moreover, a shoulder appears on the tan *δ* signal around −10 °C, which can be associated with the glass transition of the P(3HB-*co*-4HB) phase. Foregoing papers reported a *T_g_* of P(3HB-*co*-4HB) at −10 ÷ −15 °C, determined by DSC and DMA [25,26,45]. The glass transition temperature of the PLA phase is shifted to a higher temperature than in the first heating run (onset of *E*′ at 43.5 °C) mainly because of the presence of a more crystalline phase.

**Figure 5:**
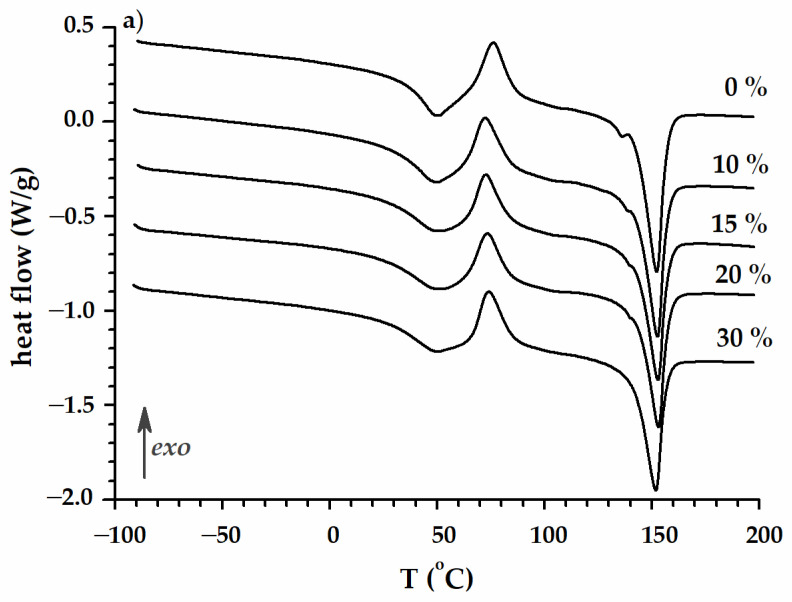

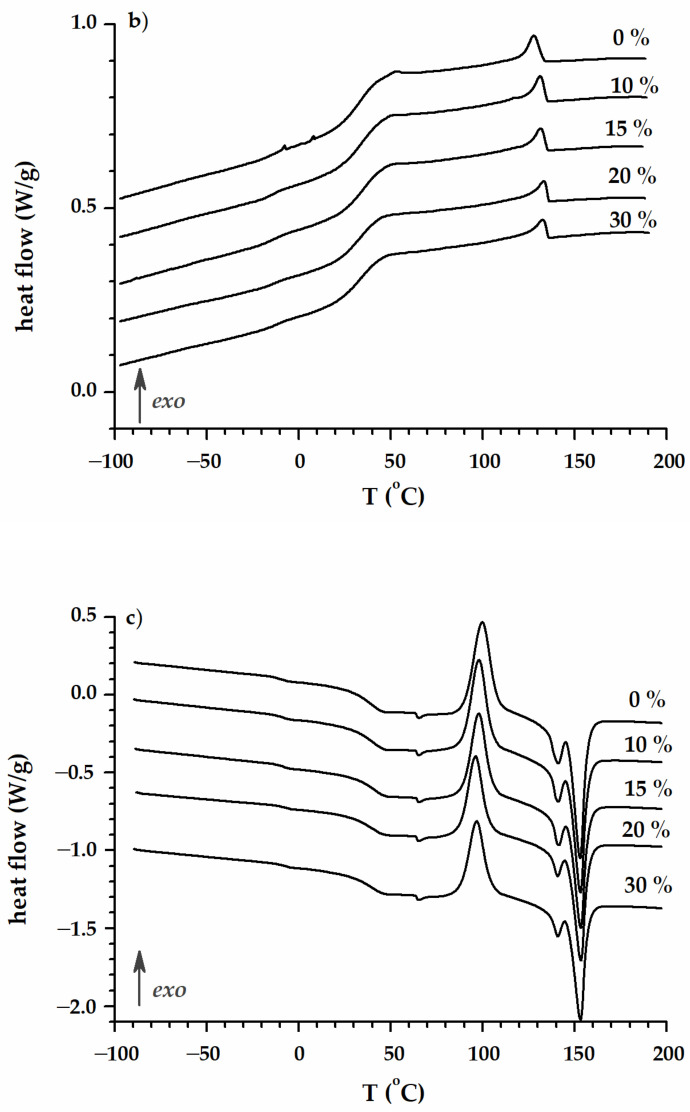
DSC curves of the neat PLA/P(3HB-*co*-4HB) and PLA/P(3HB-*co*-4HB)/biochar composites during first heating (**a**), cooling (**b**), and second heating (**c**) run.

Figure 5a–c include the DSC curves for the three runs of the DSC experiment: first heating, cooling, and second heating, respectively.

The DSC behavior during the first heating presents a glass transition that overlaps in the end part with cold crystallization. The melting temperature is around 150 °C. During cooling, a small amount of the amorphous phase crystallizes around 130 °C, and the glass transition region is registered between 32 and 35 °C. In the second heating run, two features are distinct from the first heating run: (i) the occurrence of two glass transitions: the first one around −10 °C (P(3HB-*co*-4HB)) and the second one around 35 °C (PLA); (ii) the glass transition associated with PLA and the subsequent cold crystallization are not as close as during the first heating; they are separated by more than 90 °C. The double melting peak (130–160 °C) suggests the presence of two fraction types of crystals that melt separately.

### 3.1. Electrostatic Properties

**Figure 6:**
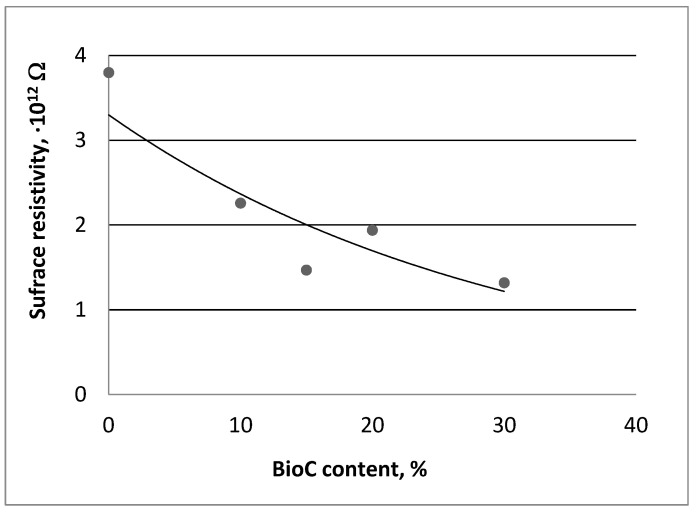
Changes in the surface resistivity value of the neat PLA/P(3HB-*co*-4HB) (100/0) and PLA/P(3HB-*co*-4HB)/biochar composites with mass ratios of 90/10, 85/15, 80/20, and 70/30.

Using fillers in composites where the matrix is a biodegradable polymer, apart from lowering the price, can introduce added values that increase the applicability of materials. The electrostatic phenomenon is not desirable in most applications, as it can lead to an unfavorable image of the product covered with dust and to the risk of explosion when such material is placed in a high-risk explosion environment [46]. The introduction of the addition of biochar to PLA/P(3HB-*co*-4HB) was intended to reduce the surface resistivity of the obtained composites and thus to use the obtained materials in special applications. The surface resistivity for neat PLA/P(3HB-*co*-4HB) and its composites as a function of different biochar content is shown in Figure 6.

The presence of biochar in the tested composites indicates a decrease in the surface resistivity value with increasing filler content. However, the decrease in surface resistivity in the case of the PLA/P(3HB-*co*-4HB) matrix is not as pronounced as in the previously described PBAT/PLA matrix [14]. Despite the clear influence of biochar on the surface resistivity of composites, it was not possible to achieve a value at which electrostatic charges could dissipate [47]. Reaching a value of surface resistivity below 1 × 10^12^ Ω makes the materials antistatic. For PLA, the value of this is in order 10^13^–10^16^ Ω, and for PBAT, it is ~10^16^ Ω [18]. When it comes to PHA, these values can vary depending on the type of monomeric unit in the polymer, and so the value in the order of 10^15^ was set for poly(3-hydroxybutyrate) (PHB) and 10^10^ for poly(3-hydroxybutyrate-*co*-3-hydroxyvalerate) (PHBV) [48,49].

Despite the high PLA content in the matrix, the reduction in surface resistivity is not as pronounced as for the PLA and PBAT matrices with increasing PLA content [18].

As can be seen, the values of surface resistivity also depend strongly on the matrix itself. This makes it possible to modify the composites not only by adding conductive filler but also by modifying the matrix.

### 3.2. Degradation Study

Modifications of previously developed biodegradable blends carried out, among others, by using various types of fillers, can also significantly change their degradation profile [14,50]. The final products made of composites containing a biodegradable matrix and biochar as a filler can be used in the packaging industry. Composites managed in this way, after fulfilling their role, should be easily selectively collected and subjected to organic recycling. The decomposition of the material during organic recycling should occur at specific time intervals [51]. Hence, there is a need to conduct research and determine possible differences in degradation profiles between the neat matrix and the composite with various filler content. Hydrolysis is one of the types of degradation that materials undergo during composting; therefore, apart from degradation studies in the compost environment, studies in water under laboratory conditions were also carried out.

**Figure 7:**
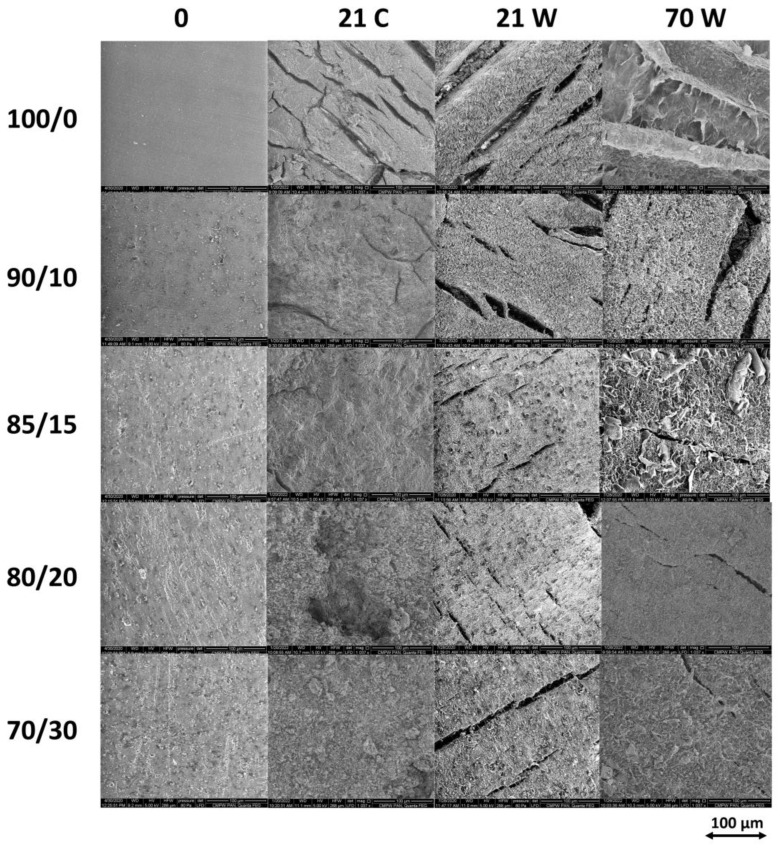
SEM micrographs of the neat PLA/P(3HB-*co*-4HB) (100/0) and PLA/P(3HB-*co*-4HB)/biochar composites with mass ratios of 90/10, 85/15, 80/20, and 70/30 before (0), after 21 days of degradation in a respirometer (21 C), and after 21 (21 W) and 70 (70 W) days of degradation in water.

Changes on the surface of composites in which the matrix is a biodegradable polymer usually indicate degradation mechanisms. The presence of cracks and pits suggests the possibility of a deeper penetration of the degradation medium into the polymer matrix. Figure 7 shows SEM micrographs of the surface of neat PLA/P(3HB-*co*-4HB) and PLA/P(3HB-*co*-4HB)/biochar composites after 21 and 70 days of degradation. 

The first row (0, Figure 7) presents changes in the surface of the PLA/P(3HB-*co*-4HB) matrix before degradation, resulting from the increase in the amount of biochar. There are clear differences in the surface morphology of samples incubated in both environments. The surface roughness increases significantly with the increase in the filler content. A thin matrix layer on the surface of the samples cracks during degradation in water for all samples and in compost (respirometer) for neat matrix and composites with low filler content (up to 15 wt%). A thin layer is formed on the surface of the samples during processing (molding to obtain dumbbell samples), as previously observed for samples containing PLA [52,53,54]. A longer time of incubation of samples in water causes the top layer to curl, starting from the edges of the cracks. Samples with a higher biochar content show more cracks on the surface and the presence of pinholes, hence the limitation of the curling effect. The observations of other researchers of greater surface changes in PBS with 5% biochar content during enzymatic degradation suggest that the incorporation of biochar may enhance the material’s susceptibility to enzymatic action [55,56].

For the samples after degradation in the compost, the increase in the filler content resulted in a significant decrease in the number of cracks on the surface and the presence of irregularities and pits. Such changes may indicate that enzymatic degradation is taking place [57]. Generally, the higher the biochar content, the more stable the polymer matrix, which is due to the highly hydrophobic nature of biochar [58], which reduces the penetration of water and moisture into the polymer matrix.

The maximum decomposition temperature, which determines the thermal stability of the material, clearly indicates the multistep nature of thermal decomposition for the investigated samples. The thermal decomposition of the polymer matrix blend components, PLA and P(3HB-*co*-4HB), occurs in other temperature ranges. The effect of biochar addition on the thermal stability of the blend components is observed as in the case of PBS [59].

The degradation of the tested materials in composting conditions may occur heterogeneously due to the specificity of the preparation of industrial composting facilities. The thermal stability of the tested materials in the area of the decomposition temperatures of P(3HB-*co*-4HB) practically does not change. In contrast, in the area of the decomposition temperatures of PLA, a decrease in this value can be seen. *T_max_* occurring in the PLA decomposition area disappears for all samples after 70 days of incubation in water, indicating a faster degradation of this blend component.

A decrease in *T_m_* and an increase in Δ*H_m_* are observed for all samples during degradation. This indicates the occurrence of the degradation of the tested materials. The increase in Δ*H_m_* is caused by the self-reorganization of shorter chains present in the samples, which increase the orderliness of the samples [60] and remain in the polymer matrix due to the lack of sample disintegration and block the release of degradation products into the environment. For samples after 70 days of incubation in water, the disintegration of the material occurred, and for samples up to 15 wt% of the filler content, a reduction in Δ*H_m_* is observed. On the other hand, for samples containing 20 and 30 wt% of the filler, despite disintegration, a further increase in Δ*H_m_* can be seen. Biochar, which is filler in the tested composites, by a higher content in the sample, may cause the retention of degradation products in the material due to its potential as an adsorbent [61].

**Figure 8:**
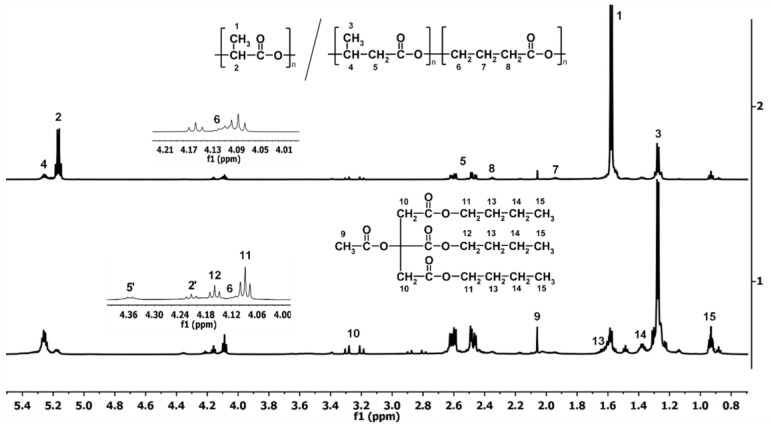
^1^H NMR spectrum of neat PLA/P(3HB-*co*-4HB) with ATBC before (2) and after 70 days of degradation in deionized water (1).

ATBC is a plasticizer that causes the decrease in PLA *T_g_*, which is responsible for the greater mobility of the chains at lower temperatures and is miscible with polymer [62]. Its presence is also important during degradation, because polymers degrading from the material cause more plasticizers to remain in it. With the progress of degradation, a decrease in PLA *T_g_* was observed, which resulted from an increase in the content of the plasticizer and a decrease in the molar mass of the degraded polymer [63]. The *T_g_* which is observed for all samples after 70 days of incubation results from the presence of an ATBC plasticizer [50]. This effect results from the increase in the mol percentage of ATBC (see Figure 8) in the samples due to the degradation of the matrix.

Changes in PLA/P(3HB-*co*-4HB) matrix composition during the hydrolytic degradation of PLA/P(3HB-*co*-4HB) wood-derived biochar composites were investigated using ^1^H NMR analysis for specimens tested after selected incubation periods. The ^1^H NMR spectrum of PLA/P(3HB-*co*-4HB) before degradation showed signals corresponding to the protons of PLA constitutional repeating units (signals 1 and 2), P3HB constitutional repeating units (signals 3–5), and signals corresponding to the protons of P4HB constitutional repeating units (signals 6–8), two components of the blend. The spectrum also showed low signals corresponding to the plasticizer (ATBC, signals 9–15) (Figure 8).

In general, neat PLA/P(3HB-*co*-4HB) and all PLA/P(3HB-*co*-4HB)-based composites showed a similar degradation course of the polymer matrix (PLA/P(3HB-*co*-4HB) blend)—the PLA component of the blend and the P4HB component of the blend’s copolymer (P(3HB-*co*-4HB)) degraded faster than 3-hydroxybutyrate (3HB) copolymer units. The addition of biochar accelerates the degradation of PLA in the PLA/P(3HB-*co*-4HB) matrix; the more biochar there is, the faster PLA decreases; in the neat polymer matrix, it degraded from 78 mol% for the initial specimen to 15 mol% for the specimen after 70 days of degradation while in composites to 1 mol%, except for the composite with 30 wt% of biochar where the amount of PLA dropped to 4 mol%. The presence of biochar accelerated the degradation of the composites probably for two reasons: (i) due to its porosity, which improved water penetration into the polymer matrix (insufficient amount of hydrophobic biochar to play a role in blocking water penetration) and (ii) because its addition causes a decrease in polymer order (decrease in the absolute value of the difference between ∆*H_m_* and ∆*H_cc_* for 100/0 and composites to 80/20; see Table 2), and the less ordered matrix degrades faster. It is generally known that hydrolysis occurs mainly in the amorphous regions of the polymer matrix, which then increases the overall crystallinity of the polymer during degradation [64]. However, the biochar content of 30 wt% increased the degradation time of the PLA component probably due to its entrapment in the matrix and higher hydrophobicity. In contrast to the PLA/PBAT wood-derived biochar composites, where mainly one component of the polymer matrix (PLA) degraded first, in PLA/P(3HB-*co*-4HB) wood-derived biochar composites, the degradation occurred throughout the entire matrix from the beginning, only at different rates of individual components, which also makes PLA degrade slower than in composites with the PLA/PBAT matrix [14].

The analysis by ^1^H NMR, based on the methine moiety of PLA and methylene moiety of P3HB, P4HB (signal 8), and ATBC (signal 10) after 21 days of degradation, indicated only slight changes (no more than 4% of PLA component and 8% of 4HB units) in the compositions of the polymer matrix investigated in the biodegradation experiment under composting conditions.

## 4. Conclusions

By carefully choosing the type and quantity of fillers and polymers in composite materials, manufacturers can effectively tailor electrostatic properties to meet specific application requirements, enhancing performance in various environments by either increasing or reducing conductivity as needed. In the case of biodegradable materials, the influence of the filler on the degradation rate may prove to be crucial when planning applications. Our research indicates the possibility of regulating electrostatic properties and additionally planning materials with a specific durability time in the environment. The developed composites, where the matrix is PLA/P(3HB-*co*-4HB) with a different biochar content, were subjected to tests of changes in surface resistivity with a change in the amount of filler and degradation tests in various environments. The obtained results indicate a clear influence of the presence of the filler on the surface resistivity, which decreases for composites with an increasing amount of biochar. However, these changes do not make it possible to achieve the value of this parameter at a level where one can talk about an antistatic material. However, it allows for broadening the applications of the obtained materials and indicates further research directions related not only to the amount of filler but also to matrix modifications. Degradation tests show differences in their profiles due to the presence of filler. In composites with lower biochar content, the effect of curling crack edges during hydrolytic degradation was not visible with higher filler content. On the other hand, incubation in the compost reduces the formation of cracks, showing the presence of pits and suggesting the occurrence of an enzymatic process [65]. The thermal properties and their changes during degradation also depend on the presence of the filler. This can be seen, for example, in the increase in melting enthalpy to 50.3 J/g after 70 days of degradation in water for samples with a biochar content above 20 wt%. This phenomenon is most likely due to the potential of biochar as an absorber. The composition of the composite matrix during degradation changes differently depending on the filler content. An increase in its amount to 20 wt% accelerates the loss of PLA from the matrix to 1 mol%, while at 30 wt%, this effect is slowed down where it reaches a value of 4 mol%. The results indicate changes occurring in the material due to the addition of filler. Its presence, however, does not block degradation processes, thanks to which it retains its biodegradability, enabling its application in environmentally friendly products. Degradation tests and previously conducted mechanical property tests indicate that the composition with 10 wt% of biochar is optimal.

## Figures and Tables

**Table 1:** The viscoelastic parameters of the composites before degradation.

PLA/P(3HB-*co*-4HB)/Biochar (Mass Ratio)	*E*′ [MPa] (Glassy Region at 0 °C)	*T_g_* [°C] ^1^ (by *E*′*_onset_*)	*T* ^2^ [°C]	*E*′ ^2^ [MPa]	*T* [°C] ^3^	*E*′ [MPa]
100/0	925	36.1	59.0	55.5	75.6	230
90/10	1400	39.2	58.5	48.1	76.6	241
85/15	1120	37.3	60.0	81.3	77.6	379
80/20	1200	41.6	59.3	76.0	78.3	318
70/30	1180	41.4	56.0	99.0	79.2	501

^1^ The glass transition temperature estimated as the onset of *E*′; ^2^ the temperature and the elastic modulus *E*′ at which the effects of cold crystallization subdue the ones of glass transition; ^3^ the temperature and the *E*′ modulus at the very beginning of the rubbery plateau.

**Table 2:** This table outlines the changes observed in the thermal properties of samples during degradation processes.

PLA/P(3HB-*co*-4HB)/Biochar (Mass Ratio)	Time [Days]	*T*_max_ [°C]	*R_600_* [%]	*T_cc_* [°C]	∆*H_cc_* [J/g ]	*T_m_* [°C]	∆*H_m_* [J/g ]	*T_gPHA_* [°C]	*T_gPLA_* [°C]
Abiotic degradation									
100/0	0	283.8/351.1	1.8	83.6	20.4	157.1	29.1	−5.9	35.9
	7	300.2/352.5	1.2	----	----	141.8	41.3	----	13.8
	14	303.7/350.0	1.0	----	-----	135.3	57.0	----	14.7
	21	301.7/344.5	1.6	----	-----	124.9	61.7	----	14.9
	70	286.5	1.7	----	-----	128.3	57.3	−21.3	----
90/10	0	286.3/341.6	9.5	86.0	20.1	160.5	27.0	−5.9	41.4
	7	295.5/341.8	8.6	----	----	146.4	37.7	----	20.2
	14	305.2/344.8	10.3	----	----	133.2	48.2	----	17.0
	21	302.6/349.0	13.1	----	-----	127.2	51.7	----	15.5
	70	289.1	30.2	----	-----	132.7	43.8	−17.7	----
85/15	0	285.9/338.8	13.6	85.7	22.1	158.8	23.7	−4.6	39.5
	7	302.9/342.7	13.5	----	----	144.7	33.6	----	19.1
	14	305.9/349.1	15.3	----	----	133.6	43.3	----	17.7
	21	303.3/351.2/	17.5	----	----	123.7	46.8	----	19.1
	70	290.0	40.4	----	----	126.6	37.7	−16.2	----
80/20	0	283.7/336.9	15.7	88.6	19.5	157.7	21.0	−5.7	39.6
	7	298.6/339.0	15.7	----	----	144.7	35.6	----	22.7
	14	306.3/347.7	18.0	----	----	135.8	44.7	----	18.5
	21	303.7/347.1	21.4	----	----	124.6	49.0	----	18.9
	70	290.0	44.9	----	----	123.4	50.3	−16.8	----
70/30	0	283.9/331.6	21.3	89.7	19.9	159.2	22.6	−5.5	41.4
	7	302.1/340.2	21.2	----	----	143.7	39.3	----	23.3
	14	306.2/341.9	24.2	----	----	135.5	44.9	----	20.4
	21	303.5/345.6	27.3	----	----	130.3	43.9	----	21.9
	70	289.9	52.4	----	----	72.1/ 118.5	60.0	−16.2	----
Degradation in compost (respirometer)									
100/0	21	285.4/327.7	2.6	----	-----	148.9	54.3	-----	29.6
90/10	21	273.6/317.6	12.3	----	-----	144.1	47.6	-----	28.1
85/15	21	290.3/336.4	14.0	----	-----	145.8	49.8	-----	29.8
80/20	21	272.2/305.3	21.2	----	------	150.1	40.1	-----	32.0
70/30	21	293.4/325.6	23.2	----	-----	145.9	45.4	-----	32.1

*T_max_*—maximum decomposition temperature, *R_600_*—residual mass at 600 °C, determined by TGA, *T_cc_*—the maximum of the exothermic peak of the cold crystallization temperature, Δ*H_cc_*—cold crystallization enthalpy, *T_m_*—melting temperature, Δ*H_m_*—melting enthalpy (first heating run, 20 °C/min), *T_g_* (second heating run after rapid cooling, 20 °C/min).

## Data Availability

The raw/processed data required to reproduce these finding is available upon request.
